# Disintegration of microtubules in *Arabidopsis thaliana* and bladder cancer cells by isothiocyanates

**DOI:** 10.3389/fpls.2015.00006

**Published:** 2015-01-22

**Authors:** Anders Øverby, Mette S. Bævre, Ole P. Thangstad, Atle M. Bones

**Affiliations:** Department of Biology, Norwegian University of Science and Technology (NTNU)Trondheim, Norway

**Keywords:** isothiocyanates, plant defense, *Arabidopsis thaliana*, bladder cancer, glucosinolate-myrosinase pathway

## Abstract

Isothiocyanates (ITCs) from biodegradation of glucosinolates comprise a group of electrophiles associated with growth-inhibitory effects in plant- and mammalian cells. The underlying modes of action of this feature are not fully understood. Clarifying this has involved mammalian cancer cells due to ITCs' chemopreventive potential. The binding of ITCs to tubulins has been reported as a mechanism by which ITCs induce cell cycle arrest and apoptosis. In the present study we demonstrate that ITCs disrupt microtubules in *Arabidopsis thaliana* contributing to the observed inhibited growth phenotype. We also confirmed this in rat bladder cancer cells (AY-27) suggesting that cells from plant and animals share mechanisms by which ITCs affect growth. Exposure of *A. thaliana* to vapor-phase of allyl ITC (AITC) inhibited growth and induced a concurrent bleaching of leaves in a dose-dependent manner. Transcriptional analysis was used to show an upregulation of heat shock-genes upon AITC-treatment. Transgenic *A. thaliana* expressing GFP-marked α-tubulin was employed to show a time- and dose-dependent disintegration of microtubules by AITC. Treatment of AY-27 with ITCs resulted in a time- and dose-dependent decrease of cell proliferation and G_2_/M-arrest. AY-27 transiently transfected to express GFP-tagged α-tubulin were treated with ITCs resulting in a loss of microtubular filaments and the subsequent formation of apoptotic bodies. In conclusion, our data demonstrate an ITC-induced mechanism leading to growth inhibition in *A. thaliana* and rat bladder cancer cells, and expose clues to the mechanisms underlying the physiological role of glucosinolates *in vivo*.

## Introduction

**Figure 1:**
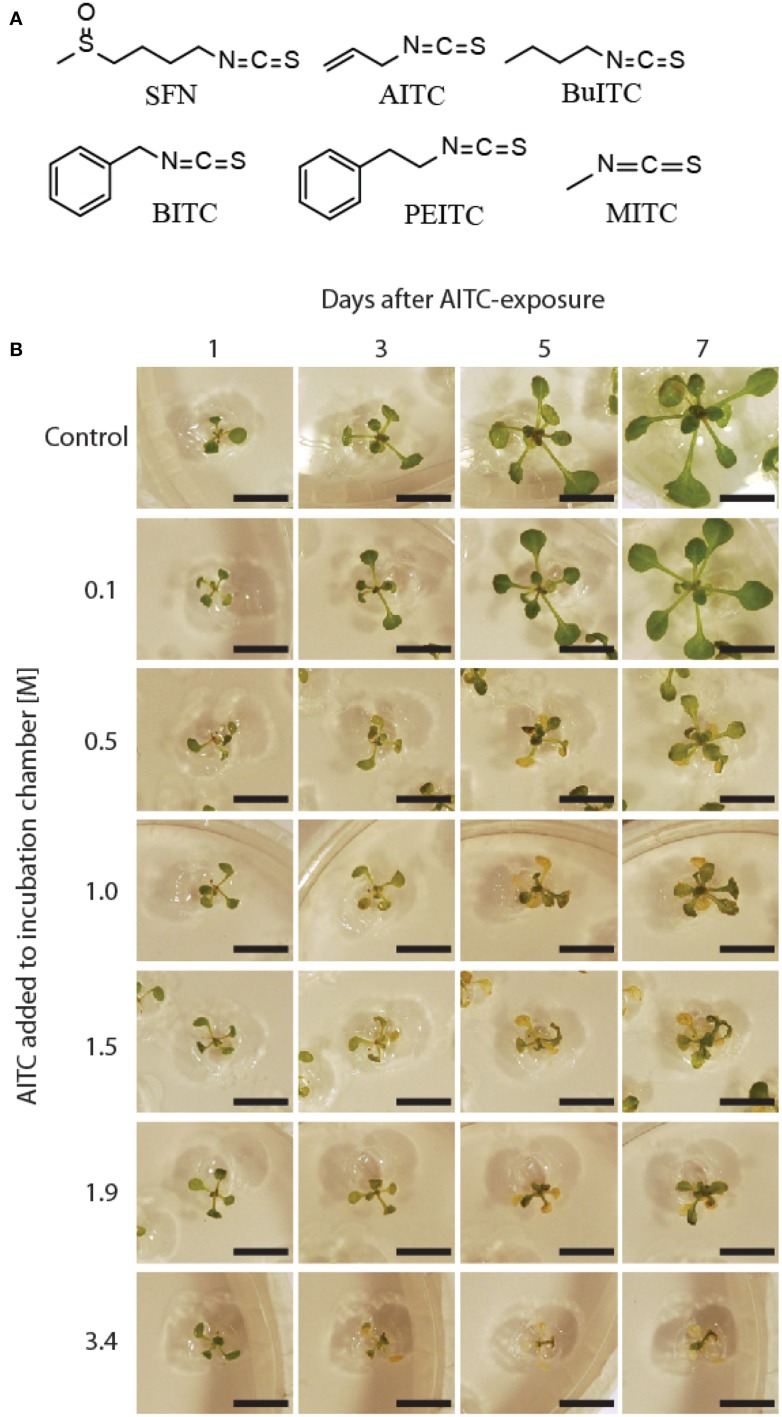
**Dose dependent inhibition of *A. thaliana* growth. (A)** Chemical structures of ITCs. **(B)** Effect of 0.1–3.4 M AITC on growth phenotype of 11-days old *A. thaliana*. Attenuated growth and bleaching of leaves were distinct from 0.5 M treatment. Bar = 1 cm.

Naturally occurring isothiocyanates (ITCs) are plant phytochemicals associated with cytotoxicity in several cell types including plant- and mammalian cells. These electrophilic compounds confer a reactive −N = C = S group linked to an R moiety influencing the potency. ITCs are found in several cruciferous edibles including mustards, broccoli, cauliflower and brussel sprouts in which they are stored as their inert precursor glucosinolate (Kissen and Bones, 2009). Glucosinolates are hydrolyzed by myrosinases upon damage of plant tissue releasing volatile ITCs causing a strong smell and a pungent taste repelling herbivores from further consuming the plant (Halkier and Gershenzon, 2006). Previous studied ITCs include sulforaphane (SFN), methyl ITC (MITC), allyl ITC (AITC), butyl ITC (BuITC), benzyl ITC (BITC), and phenethyl ITC (PEITC; Figure 1A).

ITCs are known to be potent chemopreventives possessing the ability to both prevent and inhibit tumorigenesis (Nakamura, 2009). The preventive effect has been shown through epidemiological studies in which an inverse association between the intake of ITC-producing cruciferous vegetables and development of cancer in lung, breast, colon, prostate and bladder was observed (London et al., 2000; Seow et al., 2002; Ambrosone et al., 2004; Joseph et al., 2004; Brennan et al., 2005; Tang et al., 2008). Inhibition of cancer cell growth has been shown through studies of *in vitro* cell cultures and *in vivo* studies using animal models (Zhang, 2004; Cheung and Kong, 2010). Furthermore, ITCs have been clinically trialed emphasizing their potential as chemopreventives (Shapiro et al., 2006; Cornblatt et al., 2007).

In plants, ITCs have been linked to growth inhibition involving the use of ITCs directly or by using materials and extracts containing both glucosinolate substrates and the enzymes involved in degradation (Wolf et al., 1984; Bialy et al., 1990; Vaughn and Boydston, 1997; Norsworthy and Meehan, 2005). Plants shown to be affected by ITCs include wheat, lettuce, velvet leaf, palmer amaranth and the model plant *Arabidopsis thaliana* (Wolf et al., 1984; Bialy et al., 1990; Yamane et al., 1992; Norsworthy and Meehan, 2005; Hara et al., 2010). Studies with ITCs on plants have particularly focused on weed control at which ITCs or ITC-producing entities constitute promising agents (Boydston and Hang, 1995; AlKhatib et al., 1997; Haramoto and Gallandt, 2005; Norsworthy et al., 2006). In *A. thaliana*, it has been shown that exogenously applied ITCs inhibit growth and induce bleaching of leaves (Hara et al., 2010). In addition, it was reported that AITC induces a stomatal closure in *A. thaliana* pointing toward a physiological role in the plant's defense system against water loss and pathogenic intruders (Khokon et al., 2011). More recently, Hara and colleagues reported an increased heat tolerance of *A. thaliana* by PEITC presumably through elevating the expression levels of genes encoding heat shock proteins (Hara et al., 2013).

The majority of experiments aimed at elucidating the cellular effects caused by ITCs have been conducted using mammalian cell systems, leaving the mechanism of action in plant cells rather unexplored in comparison. From studies with cancer cells, several ITC-induced chemopreventive mechanisms have been identified (Navarro et al., 2011). Although various upstream events are implicated by ITCs, it is well-established that the induction of cell cycle arrest and/or ultimately programmed cell death is important in inhibition of cancer cell growth. Mi and colleagues reported the binding of ITCs to α- and β-tubulin as an important mechanism of inducing a mitotic cell cycle arrest and subsequently apoptosis in human lung cancer cells (Mi et al., 2008). These findings have also been reported in human bladder cancer cells where tubulin bound to ITCs were ubiquitin-marked for degradation causing microtubules to disrupt (Geng et al., 2011).

In the present study we showed that ITCs disrupt microtubular filaments in *A. thaliana* contributing to the observed inhibited growth phenotype. We also confirmed the same findings in rat bladder cancer cell AY-27 leading to apoptosis suggesting that ITCs induce similar upstream events in different cell types by targeting highly conserved proteins such as tubulins. Gained insight into the ITC-induced cellular effects in plants will aid in an improved understanding of the physiological role of the glucosinolate-myrosinase system and may also lead to novel approaches to further exploration and development of ITCs as chemopreventives.

## Results

### AITC inhibits growth of *A. thaliana* in a dose-dependent manner

**Figure 2:**
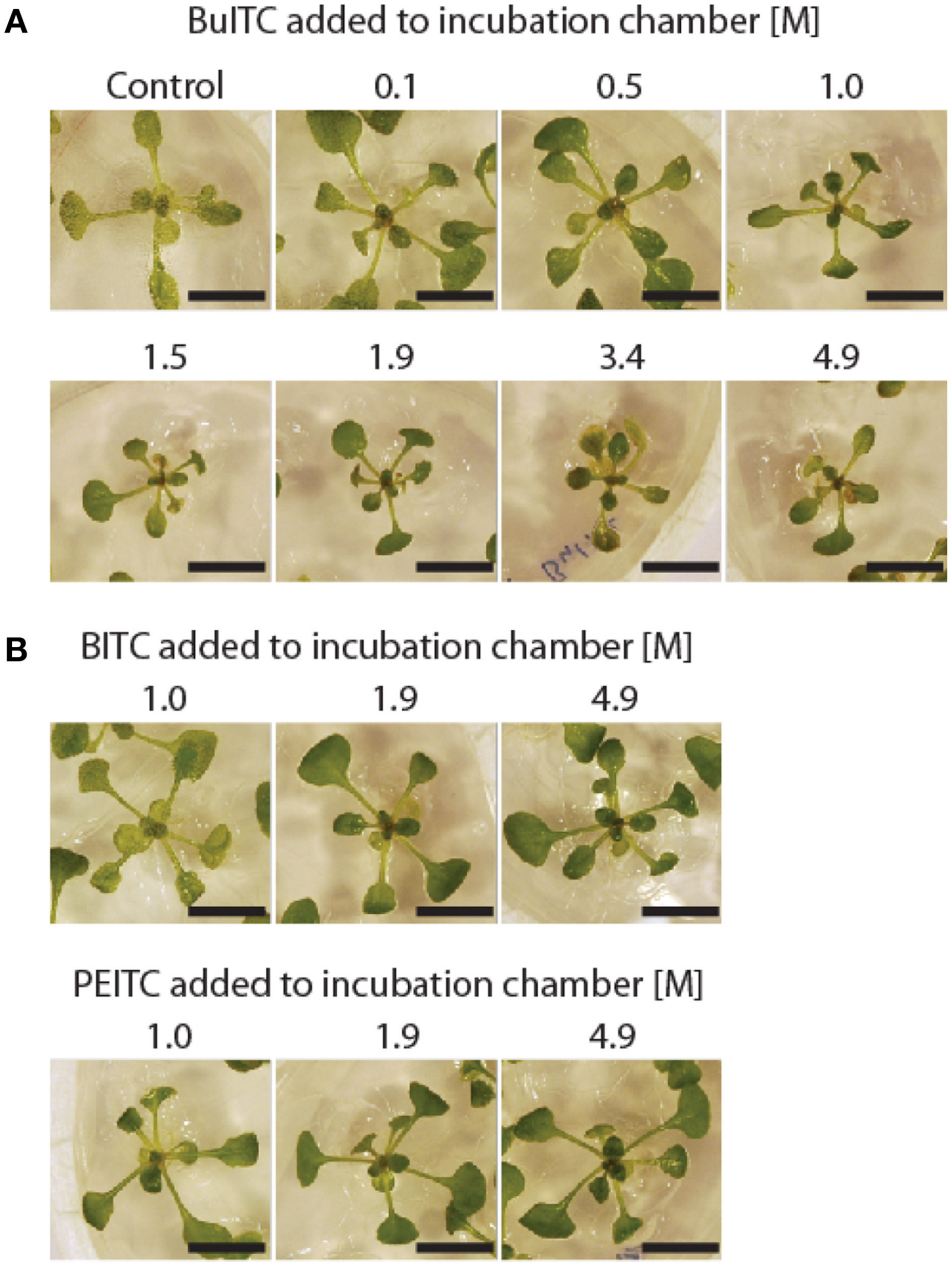
**Effect of BuITC, BITC, and PEITC on *A. thaliana* growth. (A)** Vapor-phase of BuITC inhibits growth of 10-days old *A. thaliana* when added to incubation chamber in the concentration 1.0 M. Significant reduction in plant size was observed for all concentrations 1.0–4.9 M BuITC. **(B)** Weak or no response on *A. thaliana* growth by BITC and PEITC added to the incubation chamber in concentration range 1.0–4.9 M. Bar = 1 cm.

Exposure of 11-days old plants of *A. thaliana* wild-type to the vapor-phases of AITC-dilutions in the concentration range 0.1–3.4 M inhibited growth in a dose-dependent manner (Figure 1B). The observed growth reduction was concurrent with bleaching of leaves, and visible from the exposure of 0.5 M AITC and higher concentrations. New plant tissue developed after AITC-treatment, however, did not display bleaching or miscoloring. As the extent of bleaching presumably reflects the impact caused by AITC, it is noteworthy how some exposed plants managed to recover as seen from 3.4 M exposure in Figure 1B. Lack of recovery was observed for plants exposed to vapor-phases of 4.9 and 9.7 M AITC resulting in a complete bleaching of whole plants 1 day after exposure (data not shown). A previous study has reported an ITC-induced growth inhibition and bleaching of leaves of 5-weeks old *A. thaliana* by spraying the plants with water-emulsions of ITCs (Hara et al., 2010). When employing this method, we obtained no or only weak responses in growth phenotype combined with poor reproducibility likely due to the unstable nature of ITCs in water (Ohta et al., 1995) and an uneven distribution of ITCs from spraying (data not shown). Consequently, we favored a different approach in which the volatility of AITC was exploited to generate an even atmospheric distribution of AITC in a closed growth chamber. This method yielded distinct effects on growth phenotypes from various AITC-concentrations as depicted in Figure 1B, combined with a significantly increased reproducibility. When including another aliphatic ITC, butyl ITC (BuITC), a significantly weaker response on growth was observed compared to AITC (Figure 2A). Adding the aromatic benzyl ITC (BITC) and phenethyl ITC (PEITC) resulted in no or weak responses (Figure 2B). The differences between the potential of ITCs to inhibit growth, however, are more likely explained through their different vapor pressures rather than the potentials of the ITCs to affect growth (AITC, 493; BuITC, 340; BITC, 2.0; PEITC, 1.5 Pa). Collectively, these data show that AITC induces growth inhibition and bleaching of leaves in *A. thaliana* in a dose-dependent manner and that these effects are not restricted to AITC but also induced by other ITCs e.g., BuITC.

### Disintegration of microtubular filaments in *A. thaliana* by ITCs

**Figure 3:**
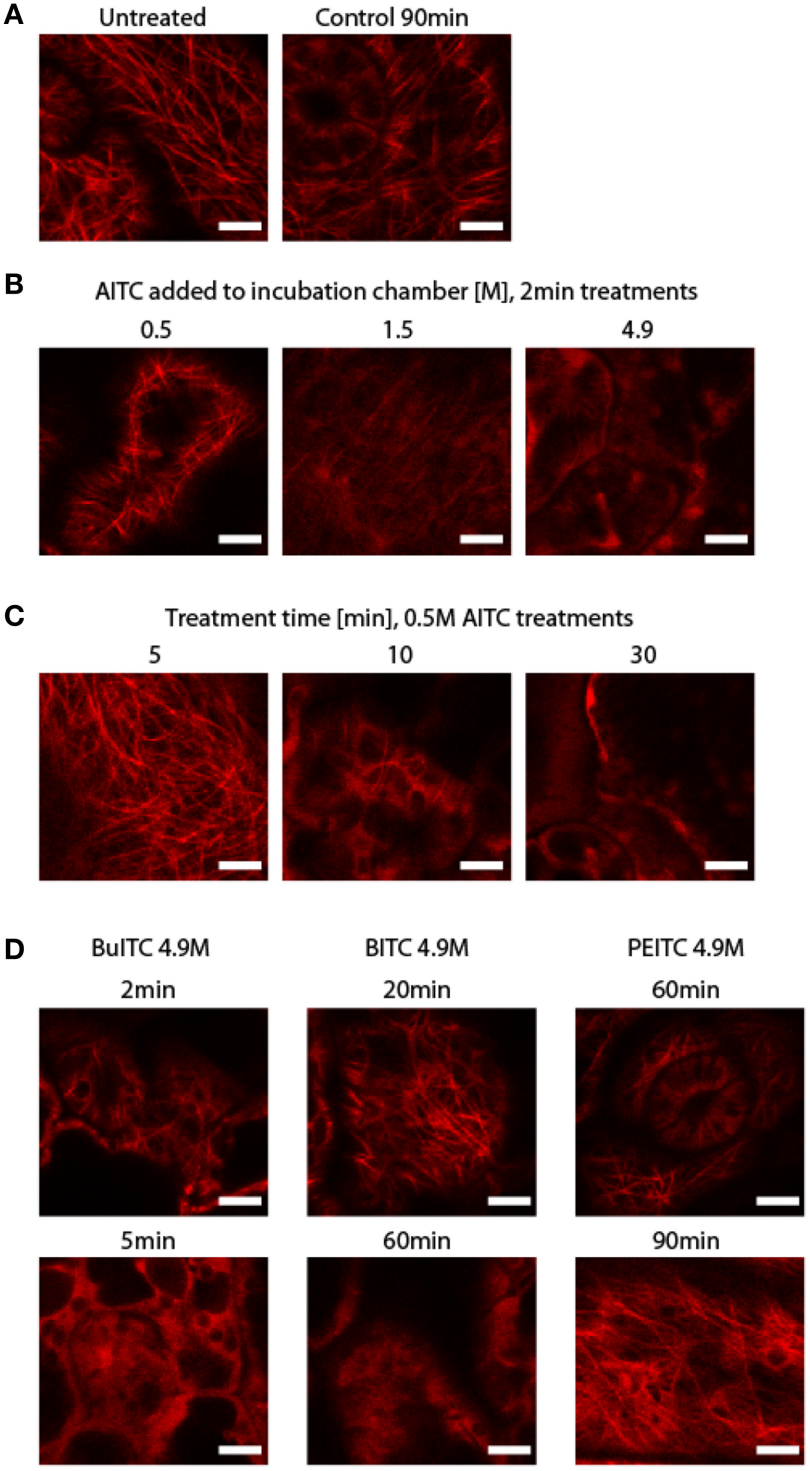
**Disintegration of microtubuli in *A. thaliana* following ITC-treatment. (A)** GFP fused N-terminally to α-tubulin renders microtubular filaments in *A. thaliana* visible through confocal scanning microscope and proved to be unaffected by 90 min of incubation in chamber with only rapeseed oil added. **(B)** Microtubuli disintegration was dose-dependent as shown by comparing plants exposed to 0.5, 1.0, and 4.9 M AITC for 2 min. **(C)** Microtubule filaments disintegration was also dependent on treatment time as shown by comparing plants exposed to 0.5 M AITC for 2, 5, 10, and 30 min. **(D)** BuITC and BITC also promoted loss of microtubule filament array in *A. thaliana* but required an increased treatment time compared to that of AITC-treatments. No effect on microtubules was observed by PEITC for up to 90 min of exposure. All plants used were 10 days old. Bar = 5 μm.

For investigating the effect of ITC on microtubules in *A. thaliana*, we employed a transgenic line expressing green fluorescent protein (GFP) fused to the N-terminal end of α-tubulin (Ueda et al., 1999). Growth phenotype of this line was shown to be unaffected by the genetic alteration when compared to wild-type following AITC-treatments (data not shown). The fusion protein rendered the microtubular filaments visible in confocal scanning microscope as shown in Figure 3A, and proved to be unaltered after 90 min of exposure to rapeseed oil without ITC. A rapid loss of the filaments was observed when plants were exposed to vapor-phase of 4.9 M AITC for 2 min (Figure 3B). Microtubule filaments were significantly reduced but still detectable when AITC-concentration was decreased to 1.5 M, and when 0.5 M AITC was applied the filaments were seemingly unaffected after 2 min of exposure (Figure 3B). These findings indicate that ITC-induced loss of microtubules in *A. thaliana* is dose-dependent. The disintegration also developed in a time-dependent fashion. Exposure of plants to vapor of 0.5 M AITC for 5 min did not reduce the filaments, but an incubation time of 10 min strongly reduced the number of filaments (Figure 3C). After 30 min of 0.5 M exposure the filaments were completely absent (Figure 3C). In order to show that the breakdown of microtubules in *A. thaliana* was not restricted to AITC we repeated the experiments with BuITC, BITC, and PEITC. As expected based on the results from the growth studies with ITCs and *A. thaliana* wild-type, an increased incubation time with these ITCs was required in order to observe an effect due to their lower vapor pressures. Exposure of 4.9 M BuITC for 2 min left a minor proportion of the filaments intact, which were lost after 5 min of treatment (Figure 3D). Adding BITC (4.9 M) to the incubation chamber did not affect the filaments after 20 min of exposure. After 60 min however, the filaments were completely lost (Figure 3D). PEITC did not show any effect on microtubules even after 90 min of exposure (Figure 3D). These results demonstrate that ITCs cause microtubules in *A. thaliana* to disintegrate in a time- and dose-dependent manner, a potential possessed by both aliphatic and aromatic ITCs.

### AITC induces transcriptional upregulation of heat shock-genes

Based on of ITCs' reactive nature and previous studies with mammalian cells, ITCs are expected to target multiple intracellular components and trigger several responses in *A. thaliana*. In order to gain more insight into the ITC-induced implications in *A. thaliana*, a microarray analysis using a custom-printed array of AITC-treated and mock-treated plants was conducted. This preliminary screen revealed a potential upregulation of genes involved in various stress responses in particular heat shock-response (data not shown). To verify this, the experiment was repeated and transcriptional regulation of a set of genes was analyzed in *A. thaliana* using quantitative PCR (qPCR). For this purpose, 11-days old plants were exposed to 0.5 M AITC for 1 h (Table S1). Indeed, the expressions of *HSP70*, *HSP90*, and *DNAJ* were induced 7.0, 9.1, and 10.8 times compared to mock-treated control plants, respectively. These chaperone-encoding genes are commonly known for being involved in securing a proper folding of intracellular proteins and are induced by several abiotic stresses (Hartl, 1996). In addition, we found that expression of *STZ* was upregulated with a relative expression of 10.9. *STZ* encodes a transcriptional regulator which expression has previously been associated with a response to drought-, saline- and cold stress (Sakamoto et al., 2004). Hara and colleagues have previously reported a response on gene expression of glutathione S-transferase (GST) in *A. thaliana* treated with a low dose of PEITC (Hara et al., 2010). This included *GSTU19* which was upregulated and *GSTF6* and *GSTF7* showing tendencies of an upregulated expression in response to ITC. However, none of these genes responded to 1 h exposure of 0.5 M AITC in our studies. We also analyzed expression of two genes encoding UDP-glucose-dependent glucosyltransferases (UGTs) due to their role in detoxification of xenobiotics. UGTs have also been reported to be upregulated in SFN-treated cancer cells (Basten et al., 2002; Messner et al., 2003). However, expressions of *UGT84A1* and *UGT75D1* were unaltered by the treatment. These findings serve as preliminary data to ITC-induced gene expression responses indicating that stress responses are likely to be triggered by ITCs in *A. thaliana*.

### ITCs inhibit proliferation and promote cell cycle arrest in rat bladder cancer cells

**Figure 4:**
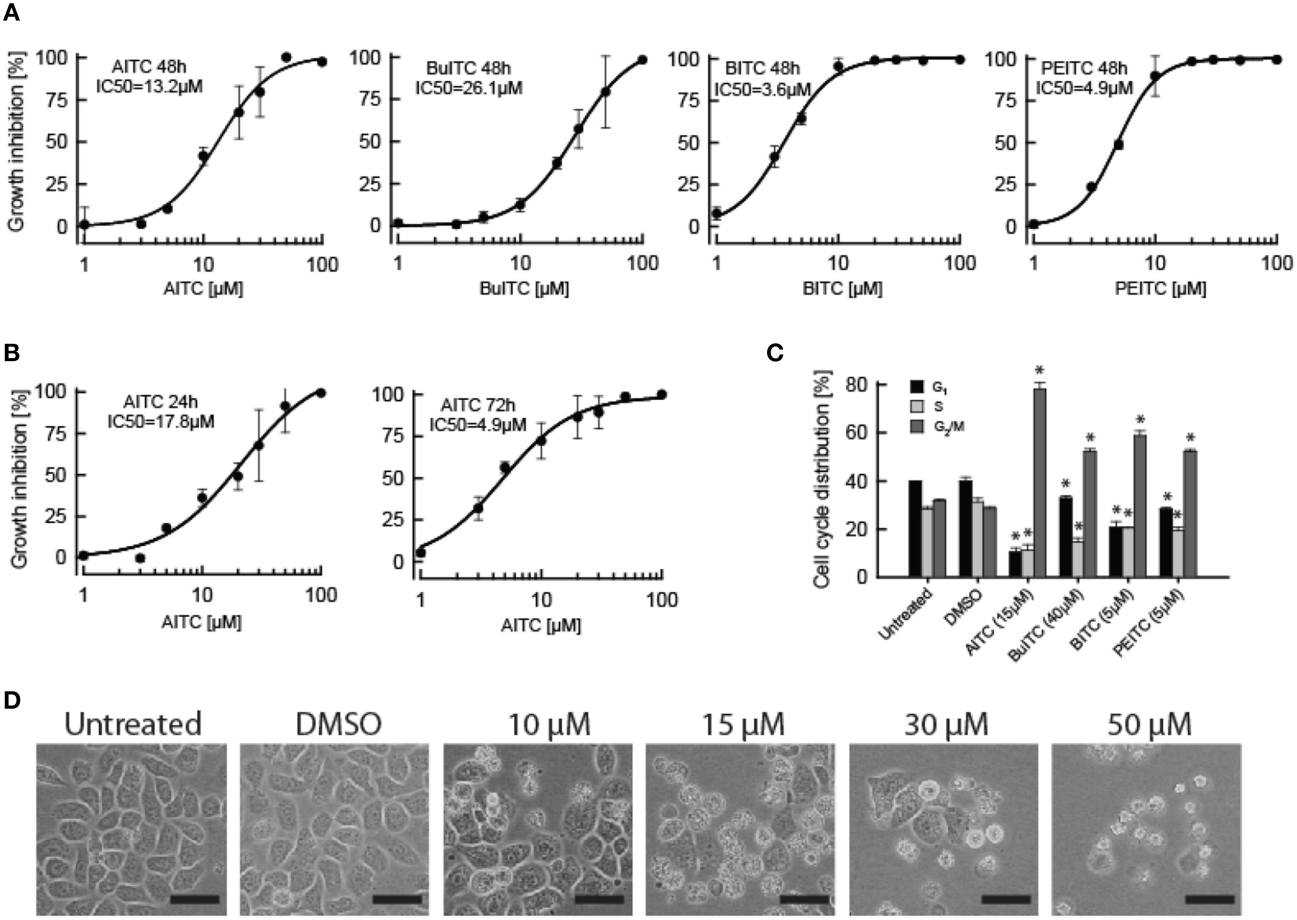
**Effects of ITCs on AY-27 proliferation, cell cycle distribution and morphology. (A)** Dose-dependent response on growth of AY-27 cells treated with AITC, BuITC, BITC, and PEITC for 48 h demonstrated using MTT proliferation assay. **(B)** A time-dependent response in AY-27 proliferation to AITC-treatment was observed when 24 and 72 h treatments were included. Logistic regression curve fits were performed to calculate the sigmoidal graphs from which the IC_50_ values were extracted. Values represent means ± s.d. of 5–6 replicates. **(C)** Effect of ITCs on cell cycle distribution of AY-27 cells treated for 24 h analyzed using flow cytometry. Concentrations used were; AITC, 15 μM; BuITC, 40 μM; BITC, and PEITC, 5 μM. Solid bars indicate cells in G_1_-phase, light-gray bars indicate cells in S-phase, dark-gray bars indicate cells in G_2_/M-phase. Values represent means ± s.d. of 3–6 replicates. The data were statistically analyzed by a two-tailed *t*-test. **(D)** Confluent AY-27 cells with increasing AITC-dosage following 24 h treatments. Bar = 50 μm. ^*^p < 0.05 (unpaired one-tail t-test).

Incubation of the rat bladder cancer cell line AY-27 with BITC, PEITC, AITC, and BuITC in the concentration range 1–100 μM for 48 h resulted in a dose-dependent inhibition of cell proliferation (Figure 4A). The IC_50_ values obtained for BITC, PEITC, AITC, and BuITC were 3.6, 4.9, 13.2, and 26.1 μM, respectively. Growth cessation also proved to be a function of treatment durability as cells incubated with AITC for 24 h yielded IC_50_ value of 17.8 μM whereas 72 h incubation resulted in an IC_50_ value of 4.9 μM (Figure 4B). Furthermore, the influence of ITCs on cell cycle distribution of AY-27 cells was analyzed with flow cytometry (Figure 4C). Treatments with 5 μM BITC and PEITC for 24 h resulted in 59 and 52% cells in G_2_/M-phase, respectively, whereas cells incubated with vehicle control had only 29% of the cells in G_2_/M-phase. Treatment with AITC or BuITC up to 15 or 40 μM, respectively, did not promote a shift in cell cycle distribution. However, incubation with 15 μM AITC and 40 μM BuITC forced 78 and 52% of the cells to reside in G_2_/M-phase, respectively. Although the AITC-concentration required for a cell cycle arrest was higher compared to the other ITCs tested, the amount of cells arrested in G_2_/M-phase induced by AITC is noteworthy suggesting that this compound may act stronger toward a cell cycle arrest than the other ITCs tested in this study. Remaining cells from AITC- and BITC-treatments were evenly distributed between G_1_- and S-phase, whereas from BuITC- and PEITC-treatments cells in G_1_-phase dominated (Figure 4C). Moreover, concurrent with dosage was the effect of ITCs on cell morphology exemplified by AY-27 cells incubated with 10, 15, 30, and 50 μM AITC (Figure 4D). The decreased number of cells with increasing AITC-concentration was accompanied with increased proportion of the cells detached from the bottom surface of the growth flasks.

### ITCs promote disintegration of microtubules in rat bladder cancer cells which subsequently enter apoptosis

**Figure 5:**
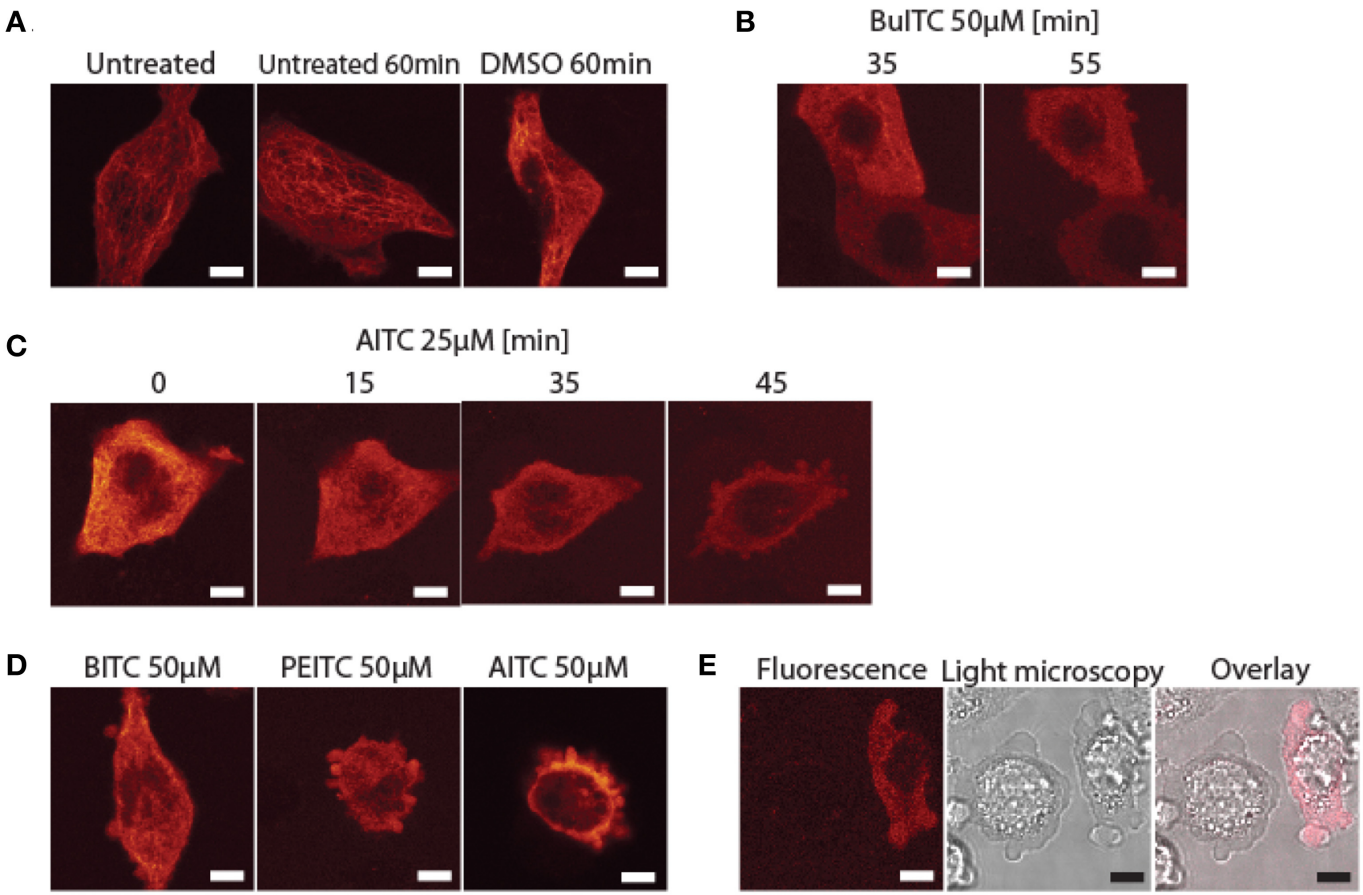
**Disintegration of microtubular filaments in AY-27 cells which subsequently enter apoptosis promoted by ITCs. (A)** GFP-tagged microtubuli in transiently transfected AY-27 cells were unaffected by a 1 h incubation under the microscope with or without solvent (0.1% DMSO) added. **(B)** AY-27 cells treated with 50 μM BuITC for 35 min displayed a significant reduction in microtubules and formed apoptotic blebs after 55 min of treatment. **(C)** AITC (25 μM) induced a significant loss of microtubules after 15 min of treatment with the subsequent formation of apoptotic bodies observed after 35 min. **(D)** Treatment of AY-27 with 50 μM BITC, PEITC or AITC for 5, 17, and 24 min, respectively, reduced microtubule filaments and induced apoptosis as seen by the formation of apoptotic bodies. **(E)** Both transfected and non-transfected cells formed apoptotic blebs as a result from BITC-treatment (50 μM). Bar = 5 μm.

To study the effect of ITCs on microtubuli in AY-27 we employed the same principal used with plants by transiently transfecting cells to express a GFP-α-tubulin fusion protein. Transfection with the plasmid PAGFP-α-tubulin mediated the expression of GFP linked N-terminally to α-tubulin in AY-27 cells. Using flow cytometry, the transfection efficiency was estimated to be 38%. During treatments, the transient transfectants were transferred from an incubator with ideal temperature and gas composition for cell growth to room temperature with normal gas composition during microscopy. Microtubule filaments appeared unaffected in both untreated- and solvent-treated cells incubated under the microscope for up to 60 min (Figure 5A). In addition, the confluence and morphology of the control cells appeared unchanged during the incubation period. Furthermore, control cells transferred back to incubator or immediately harvested and seeded out in a 75 cm^2^ growth flask (in which cells usually were maintained) displayed normal growth and morphology (data not shown). Incubation with 50 μM BuITC for 35 min significantly reduced the microtubule filaments in AY-27 (Figure 5B). After 55 min the filaments were completely absent and the cells displayed apoptotic blebs formed around the cells indicating a loss of microtubules followed by programmed cell death. This order of events appeared more distinct when AY-27 cells were treated with 25 μM AITC which after 15 min of incubation led to a significant loss of microtubular filaments (Figure 5C). The filaments were completely disintegrated after 20–25 min with the subsequent emergence of apoptotic blebs surrounding the cells after 35 min. These events merged when cells were treated with 50 μM BITC, PEITC or AITC resulting in a rapid disintegration of microtubules combined with the formation of apoptotic blebs around the cells (Figure 5D). As the cells were transient transfectants where the plasmid copy number was not controlled we hypothesized whether the metabolic burden of maintaining plasmids would affect the response to ITC-treatment. However, both fluorescing and non-fluorescing cells displayed formation of apoptotic bodies around the cells after ITC-treatment (Figure 5E). As microtubuli have been highlighted to play an important role during formation of apoptotic bodies (Moss et al., 2006) we scouted a number of apoptotic cells for fluorescing filaments without success. These data show that externally supplied ITCs may enter bladder cancer cells and disrupt microtubular filaments hindering cells to complete the G_2_/M-phase compelling cells to ultimately enter programmed cell death. The cell cycle arrest induced by ITCs as discussed above and shown in cancer cell from several sites has been further specified to be a mitotic arrest rather than a G_2_-arrest, being reasonable considering the vital role of microtubules during mitosis for segregation of chromosomes (Mi et al., 2008; Geng et al., 2011). Taken together, these data and previous reports support the fact that binding of ITCs to tubulin, causing disintegration of microtubular filaments, is an important mechanism by which ITCs lead to cell cycle arrest and apoptosis.

## Discussion

Previous studies have demonstrated that ITCs bind to tubulins, suppress the dynamic instability of microtubules and lead to their disruption as a mechanism by which ITCs induce cell cycle arrest and inhibit cell proliferation of mammalian cancer cells (Azarenko et al., 2008; Mi et al., 2008; Geng et al., 2011). In plants, ITCs have been shown to inhibit growth but information about the underlying mechanisms is missing. In the present study we confirmed the ITC-induced disruption of microtubular filaments in rat bladder cancer cells and further revealed the same mechanism in the model plant *A. thaliana*. Exposure of *A. thaliana* to vapor of 0.1–3.4 M AITC yielded a dose-dependent inhibition of growth (Figure 1B) and this was further showed to be time-related when seedlings were exposed to vapor of 0.5 M AITC for 30, 60 or 120 min (data not shown). The concurrent miscolored and bleached leaves observed following AITC-exposures (Figure 1B) indicate chlorophyll loss and are typical symptoms of oxidatively stressed plants. These traits were also observed when 5-weeks old plants of *A. thaliana* were applied with water-emulsions of MITC, AITC or PEITC in a previous report (Hara et al., 2010).

In a different study, in *A. thaliana* guard cells AITC was shown to increase the reactive oxygen species (ROS) content playing a role in stomatal closure (Khokon et al., 2011). ITC-induced ROS generation has also been demonstrated in human cancer cells subsequently to binding and conjugation of ITCs with the highly abundant redox mediator glutathione (GSH) (Zhang, 2000; Singh et al., 2005; Xiao et al., 2006; Mi et al., 2007, 2008). The conjugation reaction of GSH to ITC is carried out by the enzymes glutathione S-transferases (GSTs). Transcriptional upregulation of genes encoding GSTs has been reported to be induced by ITCs in mammalian cancer cells and has been suggested as a chemopreventive mechanism (Zhang et al., 1992; Talalay, 2000). GSTs are also present in *A. thaliana* and Hara et al. (2010) analyzed the transcript levels of a set of GSTs reporting an enhanced expression of *GSTU19* in response to PEITC (Wagner et al., 2002). Expression of *GSTU19* was not elevated by exposure to 0.5 M for 1 h in the present study. However, when exposure time with 0.5 M AITC was increased to 2 h we observed an increased expression level in all four replicates analyzed in a preliminary follow-up study. Taken together, seedlings of *A. thaliana* exposed to ITC display traits of oxidatively stressed plants which may derive from binding and/or conjugation of ITC with GSH, though further studies are required to clarify the interaction of ITC with GSH as well as the role of GSTs in ITC-exposed *A. thaliana*.

The reactivity of ITCs has been accredited the −N = C = S moiety with an R side chain dictating the specificity and potency. The presence of an aromatic component in the R group has proved to be important for the growth-inhibitory potential of an ITC supported by the present data in which PEITC and BITC exhibited a stronger inhibition of cell proliferation of AY-27 cells compared to AITC and BuITC (Figure 4A) (Jakubikova et al., 2005; Tang and Zhang, 2005; Mi et al., 2008). Our study showed activity of AITC, BuITC and BITC in *A. thaliana* (Figures 1–3) but could not range the ITCs according to order of potency as the compounds confer different vapor pressures. However, the direct application of ITC-solutions on *A. thaliana* showed that PEITC bleaches seedlings more efficiently than AITC and MITC suggesting the predominating potency of aromatic ITCs in *A. thaliana* as in mammalian cells (Hara et al., 2010).

Although influenced by the R moiety, ITCs possess the ability to bind any accessible thiol group. Consequently, after entering a cell ITCs target numerous proteins containing cysteine in addition to GSH as discussed above. In human lung cancer cells, a number of protein targets antagonized by SFN and PEITC were identified (Mi et al., 2011). A similar study has not been conducted in ITC-exposed *A. thaliana*, but several of the proteins identified by Mi et al. (2011) constitute highly conserved proteins among eukaryotes leading to an assumption that proteins targeted by ITCs in *A. thaliana* may include, but are not limited to, some of the isoforms of the proteins identified as ITC-targets in mammalian cells. We showed an upregulation in gene expressions of *HSP70*, *HSP90*, and *DNAJ* encoding chaperones involved in protein quality control and may aid in refolding proteins that are covalently modified by ITCs possessing an altered three-dimensional structure. However, many of the proteins targeted and modified by ITCs are likely to be degraded. This fate has been reported for α- and β-tubulins modified by ITCs in human lung- and bladder cancer cells presumably degraded via ubiquitination (Mi et al., 2008, 2009; Geng et al., 2011). When the binding sites of ITCs on tubulins were investigated, it was shown that the majority of the free thiol groups on cysteine residues were accessible to ITCs although influenced by the R moiety and concentration (Mi et al., 2008). Geng et al. (2011) identified cysteine residues Cys347 and Cys376 in α-tubulin and Cys12, Cys239, and Cys354 in β-tubulin from porcine cells as binding targets for AITC. Tubulins are proteins that are highly conserved among eukaryotes and amino acid sequence similarity between human and *A. thaliana* α-tubulin has been reported to 83% (Ludwig et al., 1987). Of the 21 cysteine residues in *A. thaliana* (11 in α- and 10 in β-tubulin), 16 are highly conserved in mammalian cells including the identified targets for AITC mentioned above (Ludwig et al., 1987; Marks et al., 1987). This supports an order of events induced by ITCs in *A. thaliana* involving covalent modification of α- and β-tubulins at cysteine residues rendering the tubulins incapable of polymerizing with the subsequent disintegration of microtubule filaments. It remains an open question whether the ITC-bound tubulins are degraded in *A. thaliana* or undergo a refolding. Also, studies to elucidate the binding targets of ITCs in *A. thaliana* tubulins should be undertaken to improve our understanding of this mechanism.

In summary, the present study provides evidence of an ITC-induced growth inhibition of *A. thaliana* and disruption of microtubular filaments as a mechanism contributing to the observed phenotype. This mechanism was also shown in rat bladder cancer cells suggesting that plant- and mammalian cells may share several common traits in terms of highly conserved upstream binding targets and subsequent downstream events induced by ITCs. Our findings contribute to an improved understanding of the physiological role of the glucosinolate-myrosinase system by presenting new insight into the molecular mechanisms by which ITCs act in plants. Gained knowledge about the glucosinolate-myrosinase system may in turn expose clues to the underlying mechanisms of ITCs' anti-carcinogenic activity.

## Materials and methods

### Chemicals and kits

ITCs (>95% purity), DMSO, Murashige-Skoog (MS), 3-(4,5-dimethylthiazol-2-yl)-2,5-diphenyltetrazolium bromide (MTT), RPMI-1640 (including supplements), RNase, propidium iodide and Spectrum Plant Total RNA Kit were purchased from Sigma Aldrich (Norway). Rapeseed oil was bought at a local supermarket. Primers for qPCR were ordered from Eurofins MWG (Germany). QuantiTect Reverse Transcription Kit and RNase-Free DNase Set were purchased from QIAGEN (Norway). SYBRgreen and FugeneHD transfection reagent were purchased from Roche Applied Science (Norway). Four-chambered microscopy slides were purchased from Nunc (Norway).

### *A. thaliana* lines and growth

*A. thaliana* wild-type was ecotype Col-0. The transformed *A. thaliana* Col-0 expressed GFP-tagged α-tubulin under the control of a constitutive 35S promoter (Ueda et al., 1999). Seeds of transgenic line were kindly provided by Dr. Ko Shimamoto, Nara Institute of Science and Technology, JP. Seeds of *A. thaliana* wild-type (Col-0 ecotype) and transgene line were sterilized with chlorine and ethanol before vernalized for 2 days in the absence of light. Plants were grown on MS-agar medium (MS, 2.15 g/l; sucrose, 20 g/l; agar, 6 g/l; pH 5.7) in a 16 h day/8 h night cycle at room temperature. Light intensity in growth chambers was measured to 70–80 μM/m^∧^2/s.

### ITC-exposure of *A. thaliana*

For ITC-treatment, 10–11 days old plants grown on a 9 cm petri-dish with MS-agar medium were used. The petri-dish containing the seedlings without lid was placed inside a 13 cm petri-dish containing a filter paper added 200 μl of ITC-solution diluted in rapeseed oil in the given concentrations. The 13 cm petri-dish was sealed, allowing the seedlings to be exposed to the vapor from the ITC-solution. For studying the effect of AITC on growth and microtubules, 1 h and 2–30 min exposures, respectively, were employed. When applying BuITC, BITC or PEITC, exposure durations varied from 2 to 90 min. Following exposure, seedlings were carefully picked up and transferred to a new petri-dish containing fresh MS-agar medium and grown as described above. Roots were covered with 0.1% (w/v) agarose to prevent draft. Growth was monitored by capturing pictures from above on a daily basis. ITC-solutions were always prepared right before usage.

### qPCR of AITC-treated *A. thaliana*

Using a scalpel, roots of AITC-treated *A. thaliana* wild-type plants were removed before snap-freezing the remaining plant material in liquid nitrogen. Total RNA was extracted using Spectrum Plant Total RNA Kit according to the manufacturer's protocol. During RNA extraction the samples were subjected to DNA digestion using RNase-Free DNase Set. RNA concentration in each sample was measured in duplicates using a NanoDrop 1000 (Thermo Scientific). cDNA was synthesized from 1 μg RNA using QuantiTect Reverse Transcription Kit according to the manufacturer's instructions. A Lightcycler 480 (Roche Applied Science) was used to perform qPCR with SYBRgreen in 96-well plate format according to the manufacturer's procedure. The program used for qPCR consisted of a preincubation step (95°C, 5 min), 45 amplification cycles (95°C, 10 s; 55°C, 10 s; 72°C, 10 s), and a final melting curve analysis. Correct amplicons were verified by analysis of melting curves and amplicon lengths using gel electrophoresis. Cycle threshold values were calculated using the software Lightcycler 480 Software (Roche). PCR efficiency was calculated using the software LinRegPCR. Relative expression values were calculated using the software REST 2009 from QIAGEN. At4g24550.1 (*Clathrin*), At3g25800.1 (*PP2A*), and At4g34270.1 (*TIP41-like*) were used to normalize data. Primer sequences for housekeeping genes and the genes analyzed are given in Supplementary Table S2.

### AY-27 growth and proliferation assay

Rat bladder cancer cell line AY-27 (Arum et al., 2010) was kindly provided by Dr. S. Selman (Department of Urology, Medical College of Ohio, US). Monolayer culture of AY-27 cells was maintained in 75 cm^2^ flasks with RPMI-1640 supplemented with 10% (v/v) fetal bovine serum and 100 μg/l gentamicin at 37°C and 5% CO_2_ in a humidified incubator. For proliferation assay, cells were grown in a 96-well plate starting with 5 × 10^3^ cells per well. Cells were incubated for 24 h before treated with solvent (0.1% DMSO) or freshly prepared solutions of ITCs. At ended treatments, cells were added 100 μl MTT-solution (0.5 mg/ml) and incubated for 4 h from which 50 μl was then removed. The remaining suspensions were mixed with 100 μl acidified isopropanol for 20 min before absorbance at 595 nm was measured using a spectrophotometric plate reader.

### Cell cycle analysis

For cell cycle analysis, 5 × 10^5^ cells were incubated in 25 cm^2^ flasks for 24 h before treatment. Following treatment, harvested cells were resuspended and incubated with chilled methanol (−20°C, 100%) for 15 min. Pelleted cells were resuspended and incubated with 2 ml RNase (200 μg/ml) for 45 min before pelleted and resuspended in 1.5 ml propidium iodide (50 μg/ml). After 15 min incubation at room temperature, cell cycle distribution was analyzed using a Beckman Coulter Gallios flow cytometer with 20,000 cells per sample analyzed. The software Kaluza (Beckman Coulter) was used for data handling.

### Transfection of AY-27

Plasmid PAGFP-α-tubulin (Tulu et al., 2003) was purified from overnight culture of transformed *Escherichia coli* DH5α using Maxiprep kit following the manufacturer's protocol. DNA concentration was measured using a NanoDrop 1000 (Thermo Scientific). In a 96-well plate, 1.5 × 10^4^ cells were incubated over night and medium was replaced with fresh medium. To each well, a solution of RPMI-1640 without serum added supplemented with 10 μg/ml of plasmid DNA and 4% (v/v) of FugeneHD transfection reagent was added the same volume as the volume of the medium in each well. After over night incubation, cells were washed with PBS, harvested and pooled. Transfection efficiency was determined using a Beckman Coulter Gallios flow cytometer with 5000 cells scanned for fluorescence. For confocal microscopy studies of ITC-treated transfected AY-27 cells, 8 × 10^4^ cells per well were incubated in 4-well microscopy chambers over night.

### Sample preparations and confocal scanning microscopy

From ITC-treated *A. thaliana*, a rosette leaf was excised and immediately analyzed using an inversed Leica TCS SP5 confocal microscope with a 63× NA 1.2 apochromat water immersion objective. GFP was excited with a 488 nm argon-laser and emission was detected in the range 500–590 nm. Pinhole size was 1 airy unit. Pictures captured were of 1024 × 1024 formats with pixel size of 98.6 nm. For cancer cells, a microscopy chamber with transfected AY-27 cells grown over night was inspected with the same microscope and settings as described above with the following changes: emission detection range was 495–560 nm and pictures captured were in the format 512 × 512 with pixel size 60.2 nm. All data handling was performed using the software Leica Application Suite Advanced Fluorescence Lite. Apart from occasionally minor contrast-enhancements, all pictures were exported in an unedited raw format.

### Conflict of interest statement

The authors declare that the research was conducted in the absence of any commercial or financial relationships that could be construed as a potential conflict of interest.
