# Supplementary material for: Disintegration of microtubules in Arabidopsis thaliana and bladder cancer cells by isothiocyanates
Source: Front Plant Sci. 2015 Jan 22;6:6. doi: 10.3389/fpls.2015.00006 (PMC4303138; doi:10.3389/fpls.2015.00006)
Supplement: Supplementary file 1 [file DataSheet1.DOCX]

**Supplementary table 1 Exposure of *A. thaliana* to AITC results in upregulated expression of genes encoding heat shock proteins.** Summarised results from qPCR analysis of selected genes from AITC exposed *A. thaliana* seedlings. Statistical analysis was performed using the software REST 2009. “+” depicts a significant upregulation (>1.6 relative expression) and “-” depicts a significant downregulation (<0.7 relative expression) when compared to mock-treated control. *The measurements from the qPCR analysis were close to the lower detection limit of the instrument, rendering the exact calculated expression value unreliable.

| **Gene** | **0.5 M AITC 1 h** | **Relative expression** | **Std. error** |
| --- | --- | --- | --- |
| *HSP70* | + | 7.0 | 6.2-7.8 |
| *HSP90* | + | 9.1 | 8.0-10.7 |
| *DNAJ* | + | 10.8 | 9.7-11.9 |
| *STZ* | + | 10.9 | 8.5-13.1 |
| *GSTU19* |  | 1.0 | 0.8-1.2 |
| *GSTF6** |  | n/a | n/a |
| *GSTF7* |  | 0.9 | 0.5-1.6 |
| *UGT84A1* |  | 0.7 | 0.6-0.9 |
| *UGT75D1* |  | 0.8 | 0.7-0.9 |

**Supplementary table S2.** Primers used in the qPCR analyses with their sequences presented in 5’-3’ direction.

| Gene (acquisition number) | Forward primer | Reverse primer |
| --- | --- | --- |
| *Clathrin* (At4g24550.1) | CCTTGATTGAAGAGGCTGGA | AGGGAACTCAGCGCGTATT |
| *PP2A* (At3g25800.1) | TGGCTCCAGTCTTGGGTAAG | ATCCGGGAACTCATCTTTCA |
| *TIP41-like* (At4g34270.1) | GTGAAAACTGTTGGAGAGAAGCAA | TCAACTGGATACCCTTTCGCA |
| *GSTF6* (At1g02930) | AAGCTTGGTGGCGCCGTTTG | ATGTCCTTGCCAGTTGAGAG |
| *GSTF7* (At1g02920) | CAAGGACATTGCGGGCATAG | TTAGGGCAATGAGGTCATCGCC |
| *GSTU19* (At1g78380) | GAACCCTATCCTTCCTTCTG | CTTGTTCCTCACCCTTTGTC |
| *UGT84A1* (At4g15480) | TAGTGCCAAGGGAGGAAGTG | CTCAACTCCTCTGCCTTCTC |
| *UGT75D1* (At4g15550) | GGAGTTGTGGTGGTGGATAG | ACTCCTCCGCCTTGTCTTCC |
| *HSP70* (At3g12580.1) | AAGGAGCTCGAGTCTCTTTG | TGTGTCGTCATCCATTCC |
| *HSP90* (At5g52640.1) | GCTGCTAGGATTCACAGGATG | CCTCCAACTCAGGCATATCAC |
| *DNAJ* (At2g20560.1) | CTAGGGAGATTGCTGATGTC | CTCTGGGAACGTGATCTTTG |
| *STZ* (At1g27730.1) | GCCACTACGAAGGAAACAAC | CACGGTGGCTACTGCTAACG |
